# Clinical Experience With the Use of a 675 nm Laser System With a Faster Treatment Strategy for Melasma in Indian Patients

**DOI:** 10.1093/asjof/ojag191

**Published:** 2026-10-01

**Authors:** Ruta Joshi, Ojas Kulkarni, Irene Fusco, Tiziano Zingoni, Dipak Kulkarni, Swapnil Shah

## Abstract

**Background:**

Melasma is a commonly acquired hyperpigmentation disorder.

**Objectives:**

This is a retrospective, clinical study conducted to evaluate the use of 675 nm wavelength laser treatment in Indian patients affected by facial melasma.

**Methods:**

A total of 52 patients with clinically diagnosed melasma were treated with a 675 nm laser system. All participants underwent 4 treatment sessions, each performed at 27 ± 7 day intervals. Patient's pigment reduction and skin homogeneity were assessed with photographic documentation and with the use of the modified Melasma Area and Severity Index at baseline, and 3-month follow-up after the last laser treatment. In addition, patients’ satisfaction scores were evaluated.

**Results:**

Modified Melasma Area and Severity Index score significantly decreased from 3.8 ± 2.3 at baseline to 2.8 ± 1.7 after treatment. The photographic assessment was consistent with a reduction in pigmentation following treatment. Concerning patient satisfaction scores, 13% of participants reported not being satisfied, 29% were slightly satisfied, 32% were moderately satisfied, and 26% were very satisfied with the overall treatment outcome. There were no major adverse effects reported during the treatment period.

**Conclusions:**

The application of the 675 nm laser was associated with short-term improvement to reduce pigmentary symptoms associated with facial melasma in both Indian men and women. However, the retrospective design, absence of a control group, and relatively short follow-up period limit the strength of these findings. Further prospective controlled studies with longer follow-up are needed to confirm efficacy and durability of results.

**Level of Evidence: 4 (Therapeutic):**

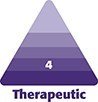

Melasma is a common acquired hyperpigmentation disorder characterized by symmetric, brown-to-gray-brown macules on sun-exposed facial areas. It disproportionately affects individuals with darker skin phototypes and is highly prevalent among women of reproductive age in South Asia, including India. Epidemiological and clinic-based studies in India have consistently identified ultraviolet radiation, hormonal influences, and genetic predisposition as major contributing factors.^1^ Because melasma is a chronic and recurrent disorder, it is challenging to manage. India's current consensus recommendations place strong emphasis on a multimodal approach that includes stringent photoprotection, topical depigmenting therapies (especially hydroquinone and triple-combination creams), and appropriate skin care practices as first-line therapy. Chemical peels and other physical modalities are recommended as second-line options after careful patient selection and skin priming.^1^

Many studies have examined laser and light-based therapies as supplemental treatments for melasma. The most commonly assessed devices include nonablative fractional platforms (eg, 1550/1540 nm), picosecond lasers, and low fluence. Q-switched Neodymium-doped Yttrium Aluminum Garnet (Nd:YAG); the Q-Switched Nd:YAG laser might be used to treat melasma because it has a longer wavelength and so does not inflame the epidermis while being efficiently absorbed by melanin cells at low dosages.^2^ Although several studies report short-term pigment reduction, results across studies are heterogeneous and the risk of postinflammatory hyperpigmentation and relapse in darker phototypes remains a major limitation. Thus, careful patient selection, conservative laser settings, and combination with topical/systemic adjuvants are commonly advocated.^3^

Among laser therapies, the 675 nm laser proved to be effective in treating melasma, acne scars, and skin rejuvenation.^4-7^ Indeed, preclinical histological investigations have demonstrated that this technology induces selective photothermal injury confined to melanin-rich regions, supporting clinical findings of significant improvement in patients with melasma.^4,8^ Several clinical published studies have been conducted to prove the efficacy and safety of 675 nm laser for melasma management also in dark-skinned individuals evaluated at up to 1-year follow-up.^8-10^

In light of these considerations, a new high-powered 675 nm laser system has been created, which can be equipped with a faster treatment modality (named Moveo) which has demonstrated effectiveness in the treatment of pigmented lesions, melasma, wrinkles, and fine lines, also in darker phototypes.^11,12^ To corroborate and confirm these findings, the purpose of this retrospective study is to present the clinical results of the 675 nm laser system integrated with the “Moveo” therapy modality to treat Indian patients affected by melasma.

## METHODS

### Study Design

This is a retrospective, clinical study conducted to evaluate the use of 675 nm laser system treatment in Indian patients affected by facial melasma. All participants provided written informed consent before inclusion, and the study adhered to the principles of the Declaration of Helsinki.

Data regarding selected potential confounders, including previous treatments, comorbidities, and occupational sun exposure, were collected when available from clinical records. All patients were instructed to adhere to strict photoprotection and posttreatment care.

### Patients and Inclusion Criteria

A total of 52 patients with clinically diagnosed melasma were enrolled. Melasma diagnosis was established clinically by experienced dermatologists based on the presence of symmetric hyperpigmented macules on sun-exposed facial areas. The study population consisted of 94% females and 6% males, with a mean age of 44.2 ± 8.2 years. According to the Fitzpatrick classification, 61% of participants had Phototype III and 39% had Phototype IV.

The mean disease duration was 4.4 ± 1.7 years. Among the participants, 61% had not undergone any previous treatment, whereas 39% had received previous therapies for melasma. A positive family history was reported in 65% of cases, whereas 35% denied familial predisposition.

Associated comorbidities included thyroid disorders, anemia, and other hormonal or systemic conditions.

Regarding occupational exposure, patients were categorized based on their predominant level of sun exposure. Specifically, 47% of participants had predominantly indoor activities with minimal occupational sun exposure, whereas 53% were engaged in occupations associated with variable or higher levels of sun exposure.

### Exclusion Criteria

Patients with a personal or family history of skin cancer, hypersensitivity to light, anticoagulant or immunosuppressive medication use, and pregnancy were excluded.

### Study Device

All patients were treated with the Red Touch PRO laser system (DEKA M.E.L.A., Calenzano, Italy), which has a 675 nm wavelength. A fractional laser beam is transmitted through the scanner, generating micro-denaturation regions (DOTs) of around 0.7 mm in size. The scanner can be fitted with a contact sensor to further improve patient safety, and it has a cooling system (the handpiece's tip has a sapphire window that can be cooled down to 5 °C) to protect the epidermis.

There are 2 modes of operation for Red Touch PRO: Standard (STD) and Moveo. Both modalities were chosen and assessed for this investigation.

In STD mode, the handpiece must be positioned perpendicular to the skin surface and held in place until the system has completed the entire 13 × 13 mm scanning area. Afterwards, the handpiece should be repositioned so as to ensure that the scanning areas do not overlap.

In Moveo mode, users must move perpendicular to the axis of the scan line because the scanner only conducts a single line of DOTs up to 13 mm long. More treatment efficiency is achieved with this modality because it enables the operator to move more quickly than in STD mode and produces a line of DOTs on the skin.^11^

### Pretreatment Care

The patient must avoid sun exposure for at least 4 weeks before therapy in order to prevent issues after treatment. They must also refrain from taking aspirin for a few days before treatment in order to prevent purpura.

To eliminate any surface debris that could absorb the laser energy, the treated areas must be shaved and cleaned with a solution of mild soap and water before the procedure. Oil, lotions, deodorant, and cosmetics are not permitted in the treated area. It is advised that the patient refrain from wearing makeup for 2 days before the procedure as a precaution.

To ascertain the appropriate values for the treatment parameters, a skin test must be conducted on the same area to be treated. The emission settings must be chosen based on the phototypes of the patients and the intended course of treatment. To effectively cool the skin's outermost layer and guarantee that the right amount of fluence is applied to the skin, a thin layer of transparent-uncolored aqueous gel must be applied between the skin and the sapphire. The handpiece must be in close contact with the skin for at least a few seconds before laser emission starts. No topical or local anesthesia was required during the procedure. Patient comfort was maintained throughout the treatment thanks to the integrated contact cooling system.

### Laser Monotherapy Protocol

All participants underwent 4 treatment sessions, each performed at 27 ± 7 day intervals.

In the first part of the session, the STD mode was applied using the following parameters: power of 3 W, pulse duration of 75 ms, spacing of 1500 μm, energy per DOT 225 mJ, and fluence of 58 J/cm^2^. Subsequently, the Moveo mode was employed, consisting of a single pass at a power of 4 W, energy per DOT 200 mJ, fluence of 52 J/cm^2^, and emitted DOTs of 2500, applied to both cheeks.

Regarding treatment, Moveo mode was specifically applied to the cheeks as the most commonly and prominently involved areas. The spot size (DOT) was ∼0.7 mm. The clinical endpoint was the appearance of mild and transient erythema without epidermal disruption.

### Posttreatment Care

Patients were instructed to apply moisturizer and sunscreen.

### Evaluation Parameters

Clinical evaluation was performed at baseline and at 3-month follow-up after the last laser treatment. Clinical evaluation of patient's pigment reduction and skin homogeneity was assessed with photographic documentation under standardized lighting conditions and with the use of the modified Melasma Area and Severity Index (mMASI). The mMASI was used as a quantitative measure of melasma severity. This validated scoring system evaluates 4 facial areas (forehead, right malar region, left malar region, and chin) by assessing the extent of involvement (scored from 0 to 6) and the darkness of pigmentation (scored from 0 to 4).

The final score was calculated using a weighted formula based on the relative surface area of each region, with a total score ranging from 0 to 24, where higher scores indicate greater disease severity.^13^

In addition, patients’ satisfaction scores were evaluated. Patients’ satisfaction was assessed using a 4-point Likert scale (not satisfied, slightly satisfied, moderately satisfied, and very satisfied). This measure was not based on a validated questionnaire but was used to provide a pragmatic assessment of patient perception in a real-world clinical setting.

Satisfaction data were collected during follow-up visits and recorded by the treating physician. Because of the retrospective nature of the study, no blinding was implemented.

A responder analysis was performed based on the percentage reduction in mMASI score from baseline to follow-up.

Safety and tolerability were assessed throughout the treatment period and at follow-up visits. Adverse events were categorized as major (eg, scarring, persistent dyspigmentation, and infection) or minor (eg, transient erythema, edema, and mild discomfort).

### Statistical Analysis

Statistical analysis was performed using standard descriptive methods. Continuous variables are reported as mean ± standard deviation, whereas categorical variables are expressed as percentages.

Changes in mMASI scores before and after treatment were analyzed using a paired Student's *t*-test. A *P*-value of <.05 was considered statistically significant.

## RESULTS

The data were retrospectively collected and analyzed. The mean mMASI score significantly (*P* < .001) decreased from 3.8 ± 2.3 (95% CI, 3.0-4.6) at baseline to 2.8 ± 1.7 (95% CI, 2.1-3.4) after treatment (Figure 1). This reduction of ∼1.0 point was statistically significant according to the paired Student's *t*-test. Although statistically significant, the magnitude of change in mMASI score was modest, and its clinical relevance should be interpreted with caution. The observed improvement suggests a potential benefit of the treatment, indicating a meaningful improvement in melasma severity following the treatment protocol. The median mMASI decreased from 3.4 at baseline to 2.5 after treatment, confirming a consistent reduction across the study population.

**Figure 1.**
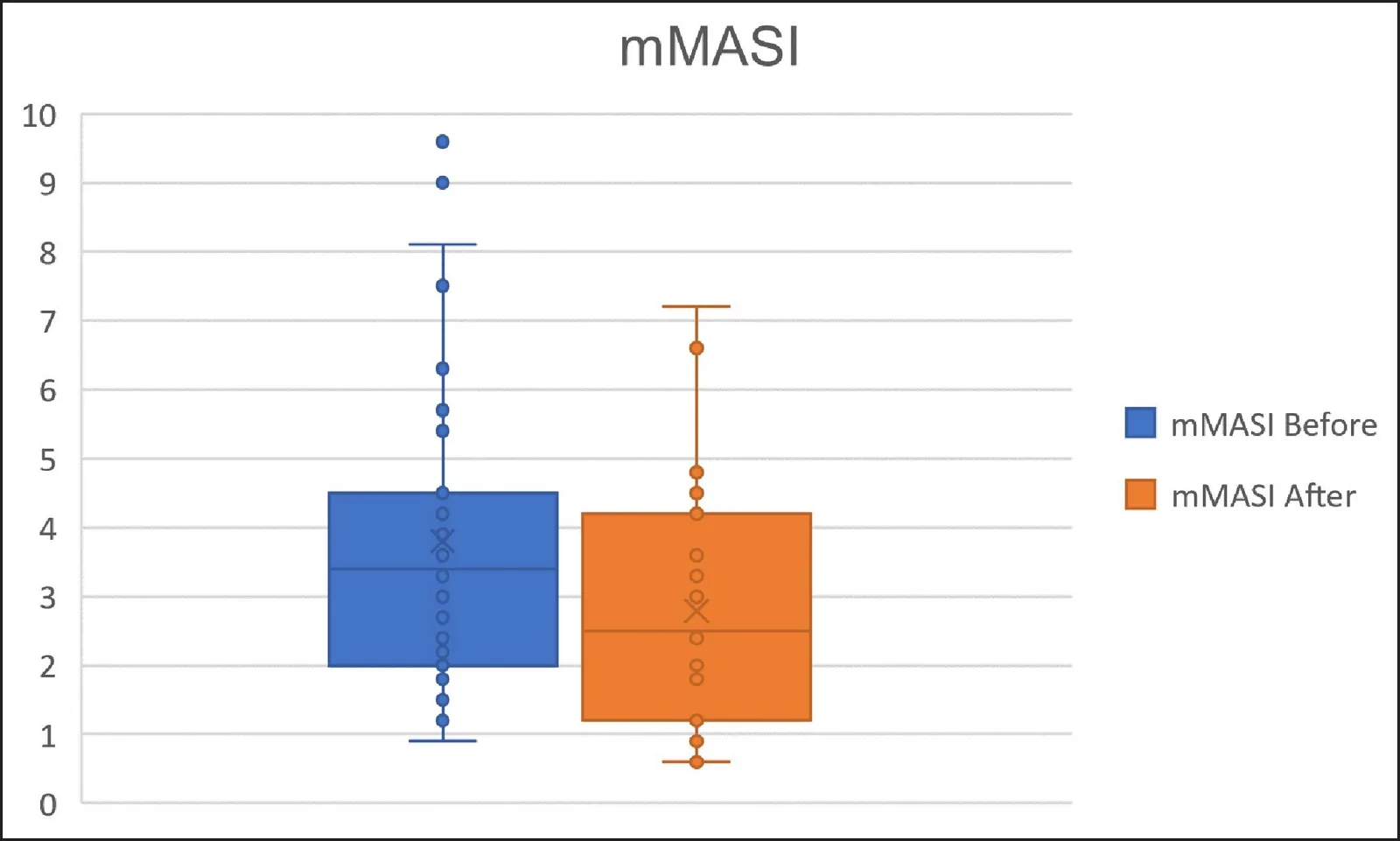
Graphical representation of the mean modified Melasma Area and Severity Index (mMASI) score.

Concerning responder analysis, most patients experienced some degree of improvement, with 39% showing 0% to 25% improvement, 35% showing 25% to 50% improvement, 16% showing 50% to 75% improvement, and 3% showing >75% improvement; 6% showed no improvement (Figure 2). The mean percentage improvement was 24.5%, whereas the median percentage improvement was 25.0%.

**Figure 2.**
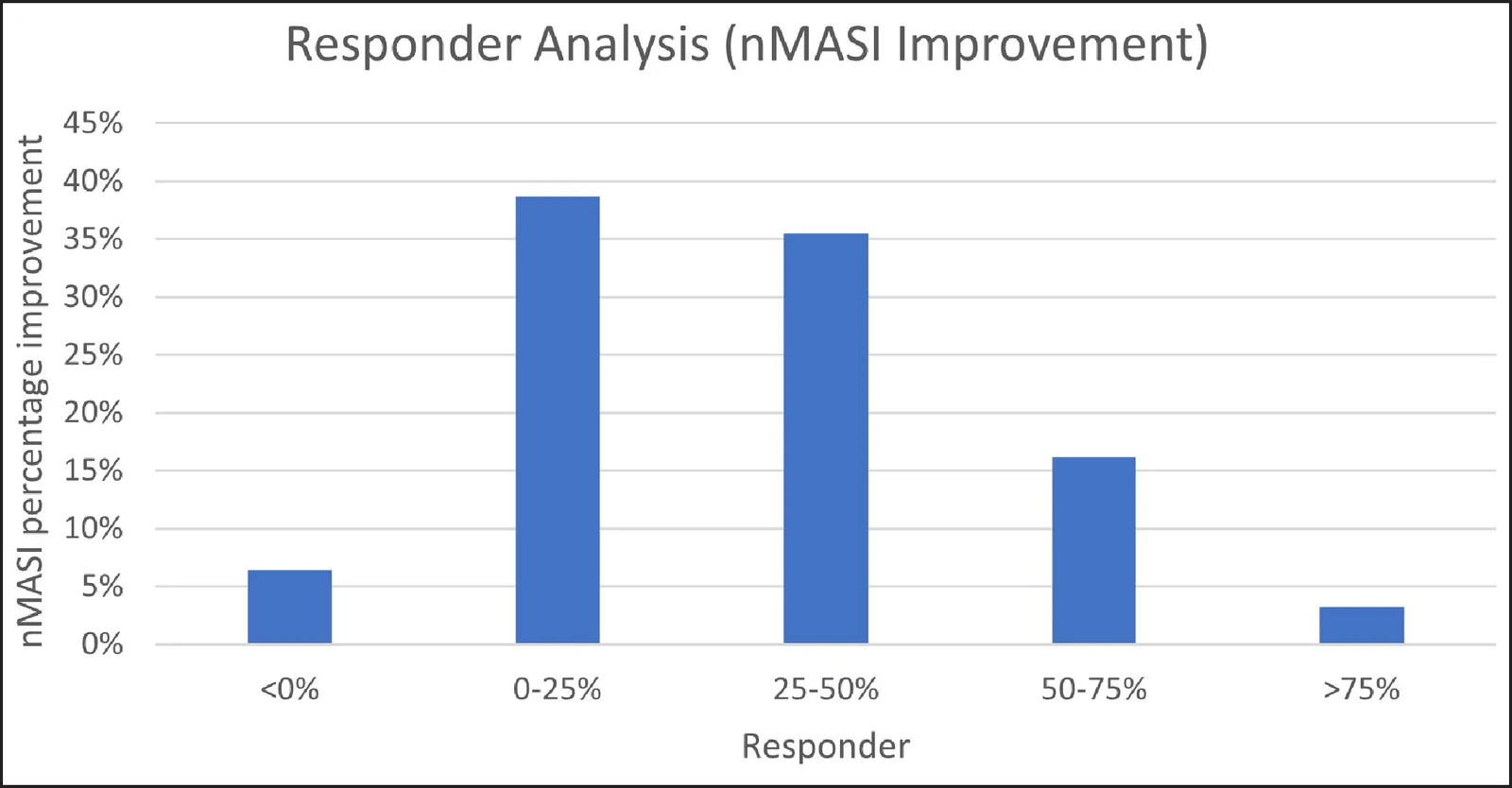
Responder analysis based on percentage improvement in modified Melasma Area and Severity Index (mMASI) score. Overall, 39% showed 0% to 25% improvement, 35% showed 25% to 50% improvement, 16% showed 50% to 75% improvement, and 3% showed >75% improvement; 6% showed no improvement.

The photographic assessment was in line with the quantitative mMASI score reductions, corroborating the effectiveness of laser therapy. Some relevant clinical cases are shown in Figures 3-9. Concerning patient satisfaction scores, 13% of participants reported not being satisfied, 29% were slightly satisfied, 32% were moderately satisfied, and 26% were very satisfied with the overall treatment outcome (Figure 10).

**Figure 3.**
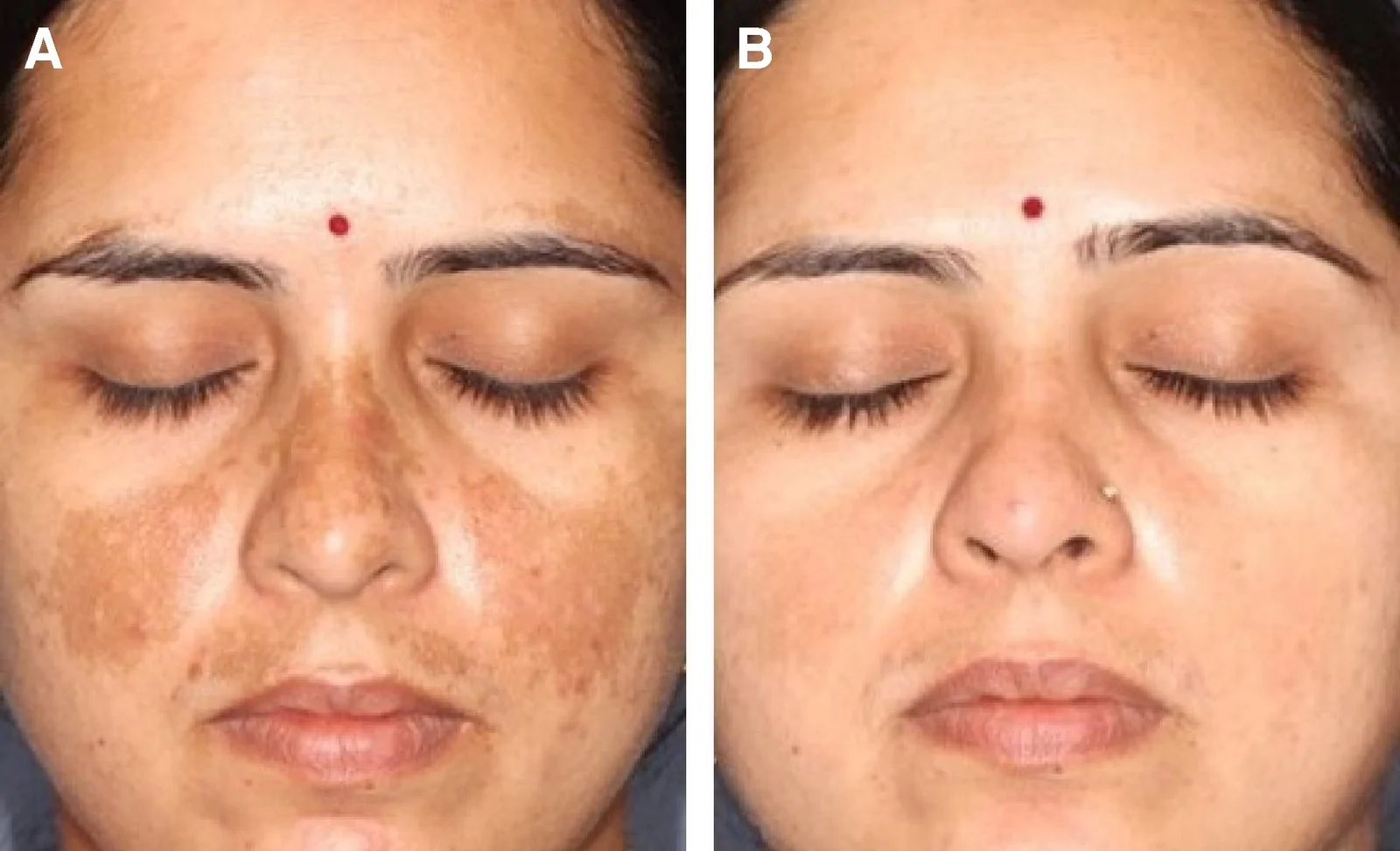
Frontal view of a 38-year-old Indian female patient affected by facial melasma (A) before and (B) 3 months follow-up after laser treatment.

**Figure 4.**
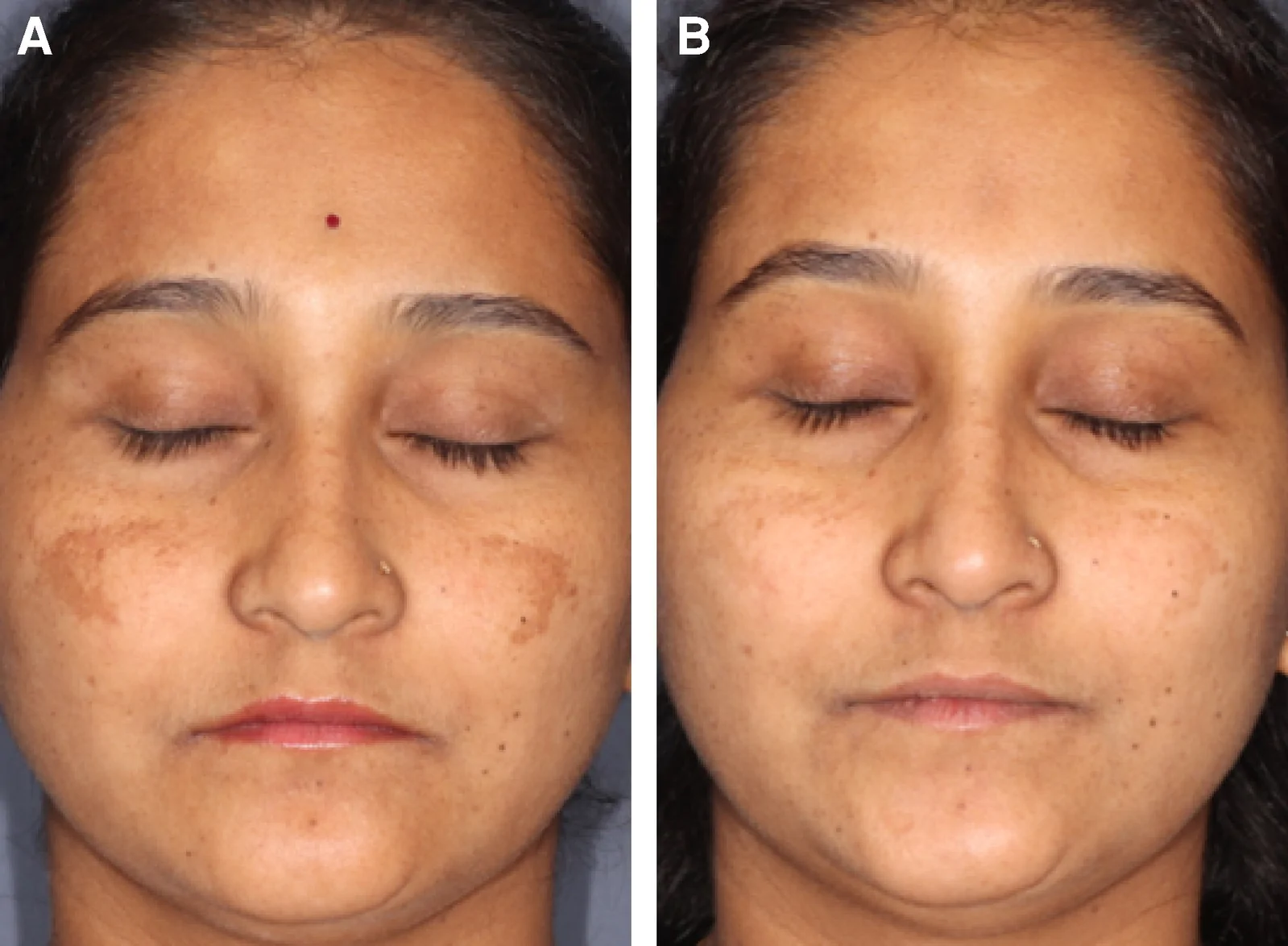
Frontal view of a 39-year-old Indian female patient affected by facial melasma (A) before and (B) 3 months follow-up after laser treatment.

**Figure 5.**
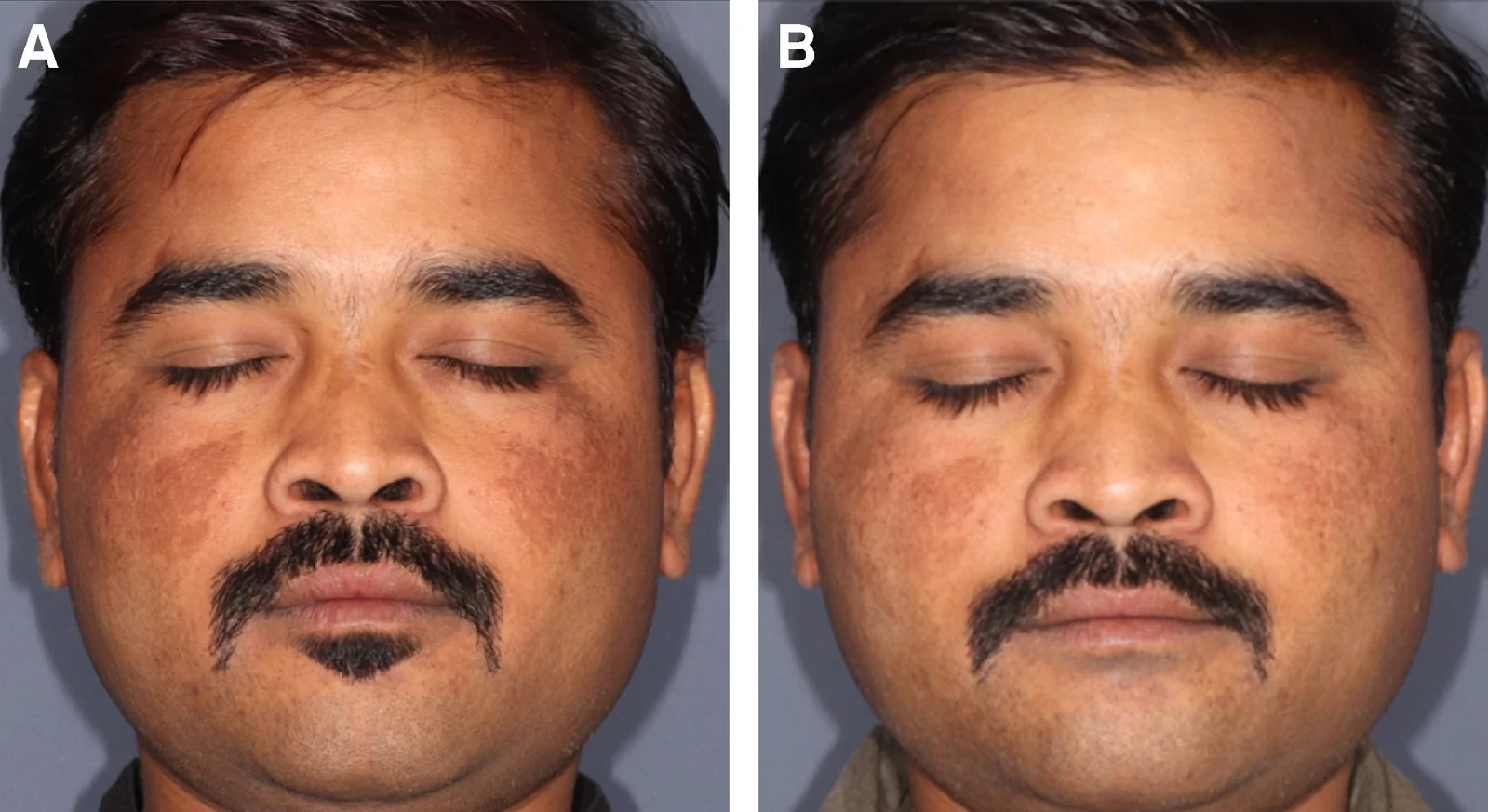
Frontal view of a 43-year-old Indian male patient affected by facial melasma (A) before and (B) 3 months follow-up after laser treatment.

**Figure 6.**
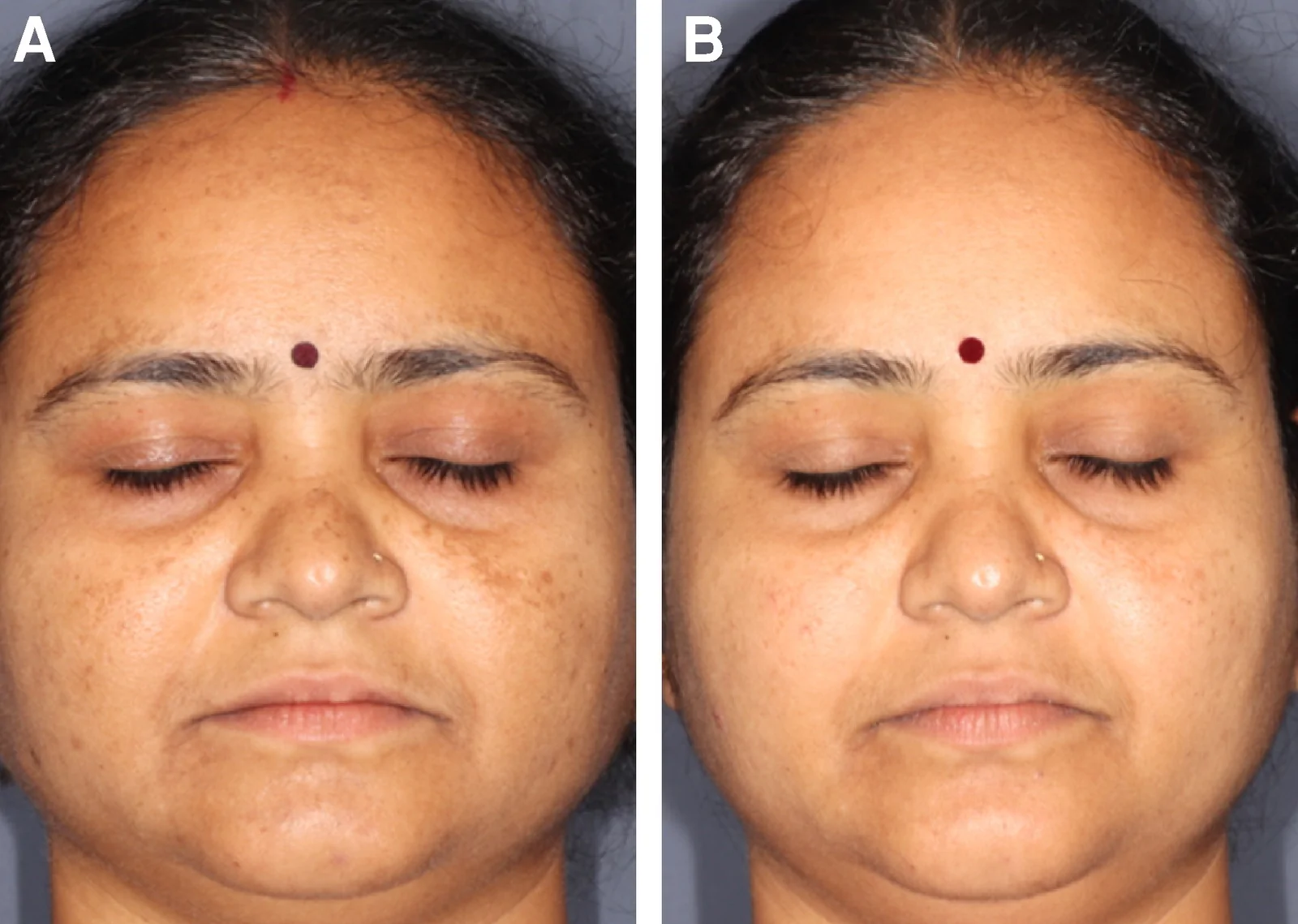
Frontal view of a 41-year-old Indian female patient affected by facial melasma (A) before and (B) 3 months follow-up after laser treatment.

**Figure 7.**
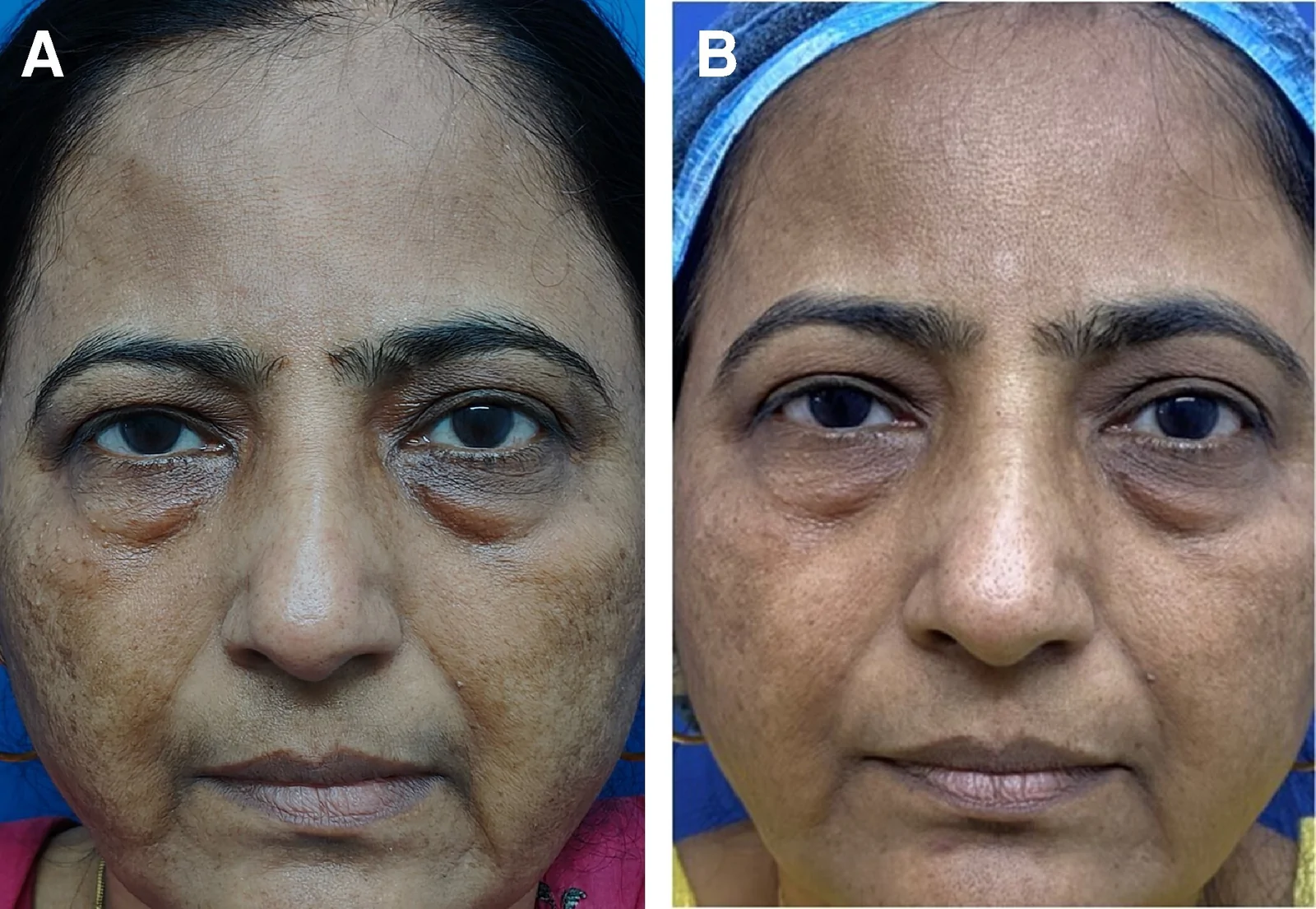
Frontal view of a 57-year-old Indian female patient affected by facial melasma (A) before and (B) 3 months follow-up after laser treatment.

**Figure 8.**
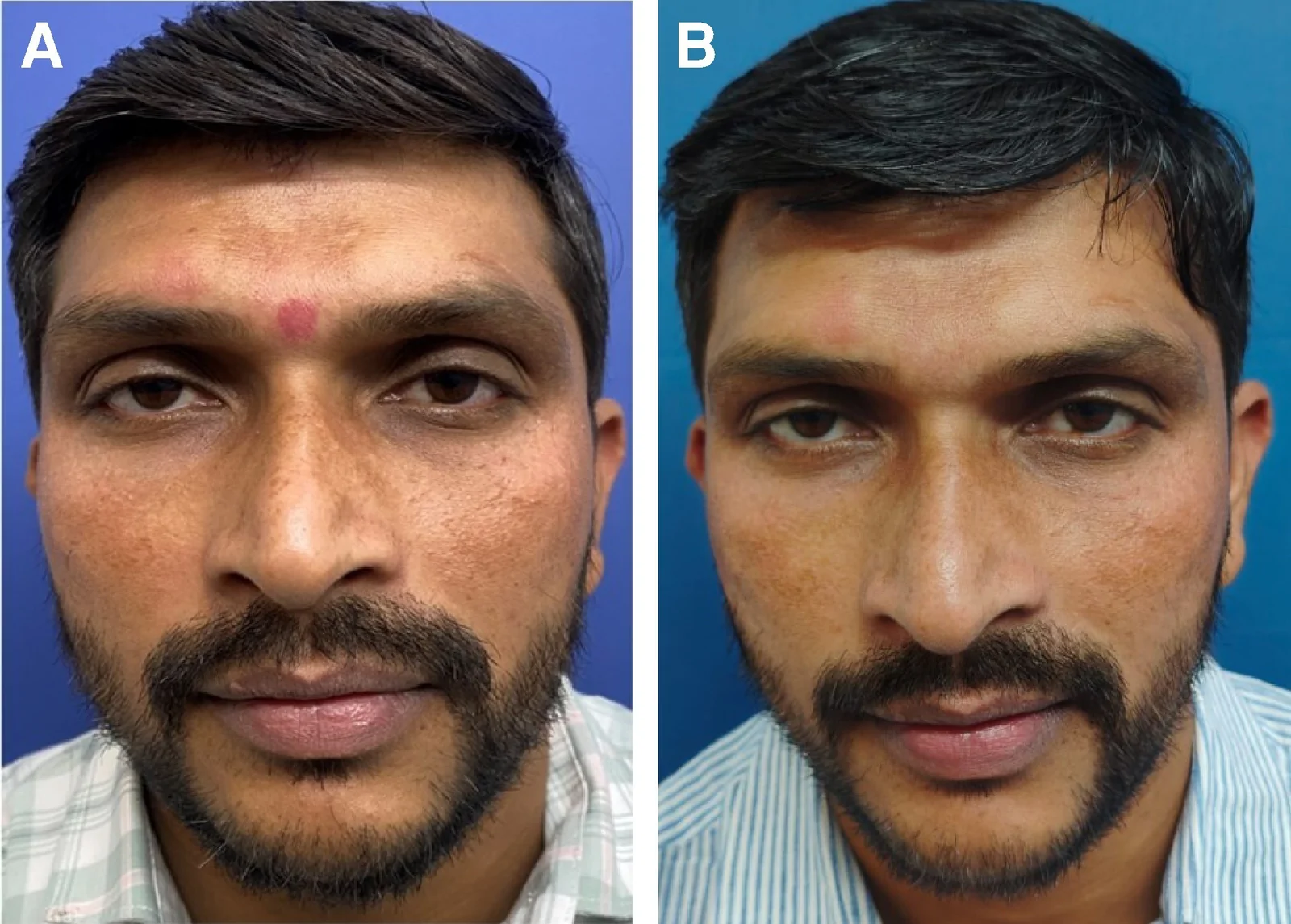
Frontal view of a 35-year-old Indian male patient affected by facial melasma (A) before and (B) 3 months follow-up after laser treatment.

**Figure 9.**
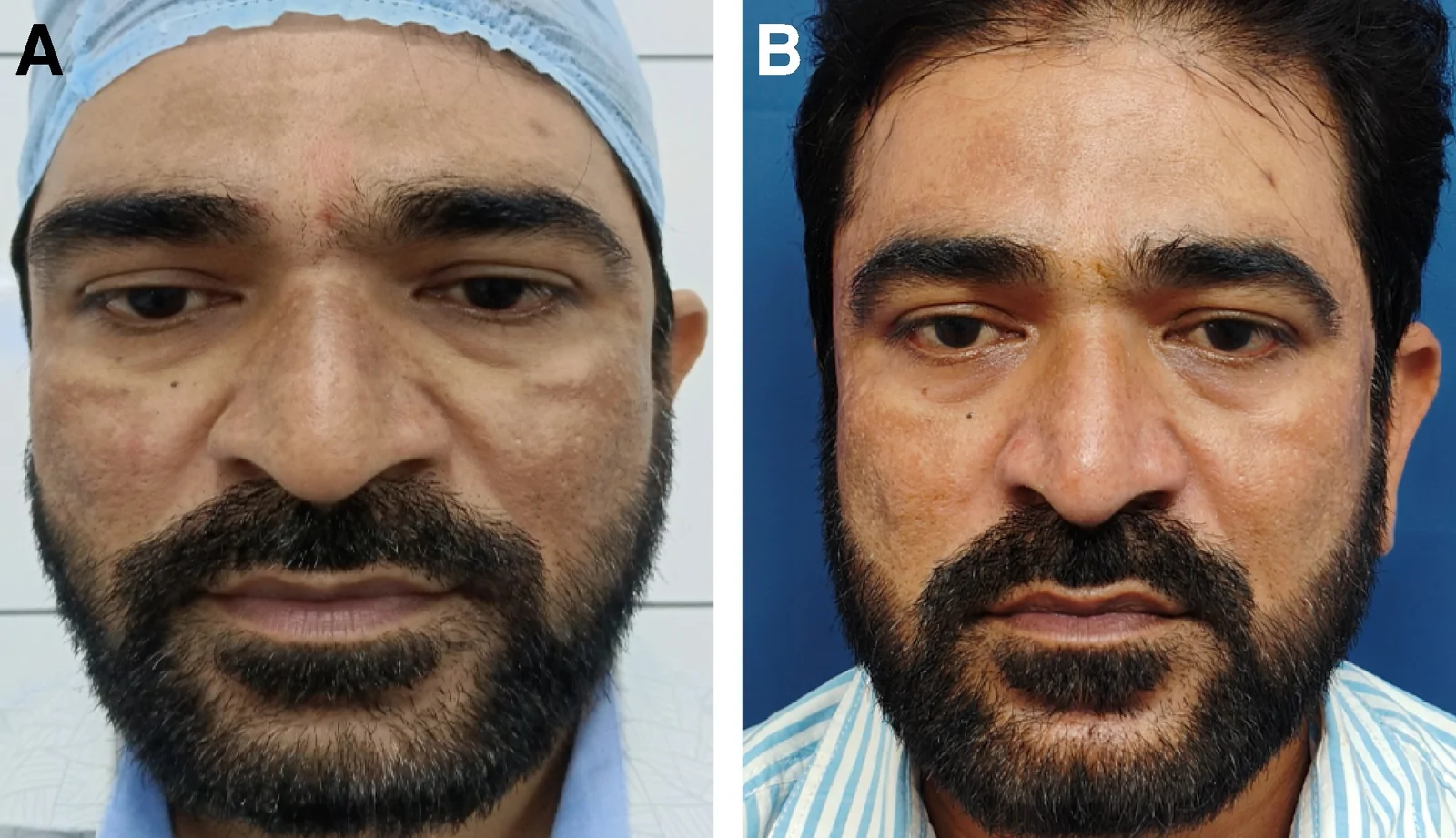
Frontal view of a 43-year-old Indian male patient affected by facial melasma (A) before and (B) 3 months follow-up after laser treatment.

**Figure 10.**
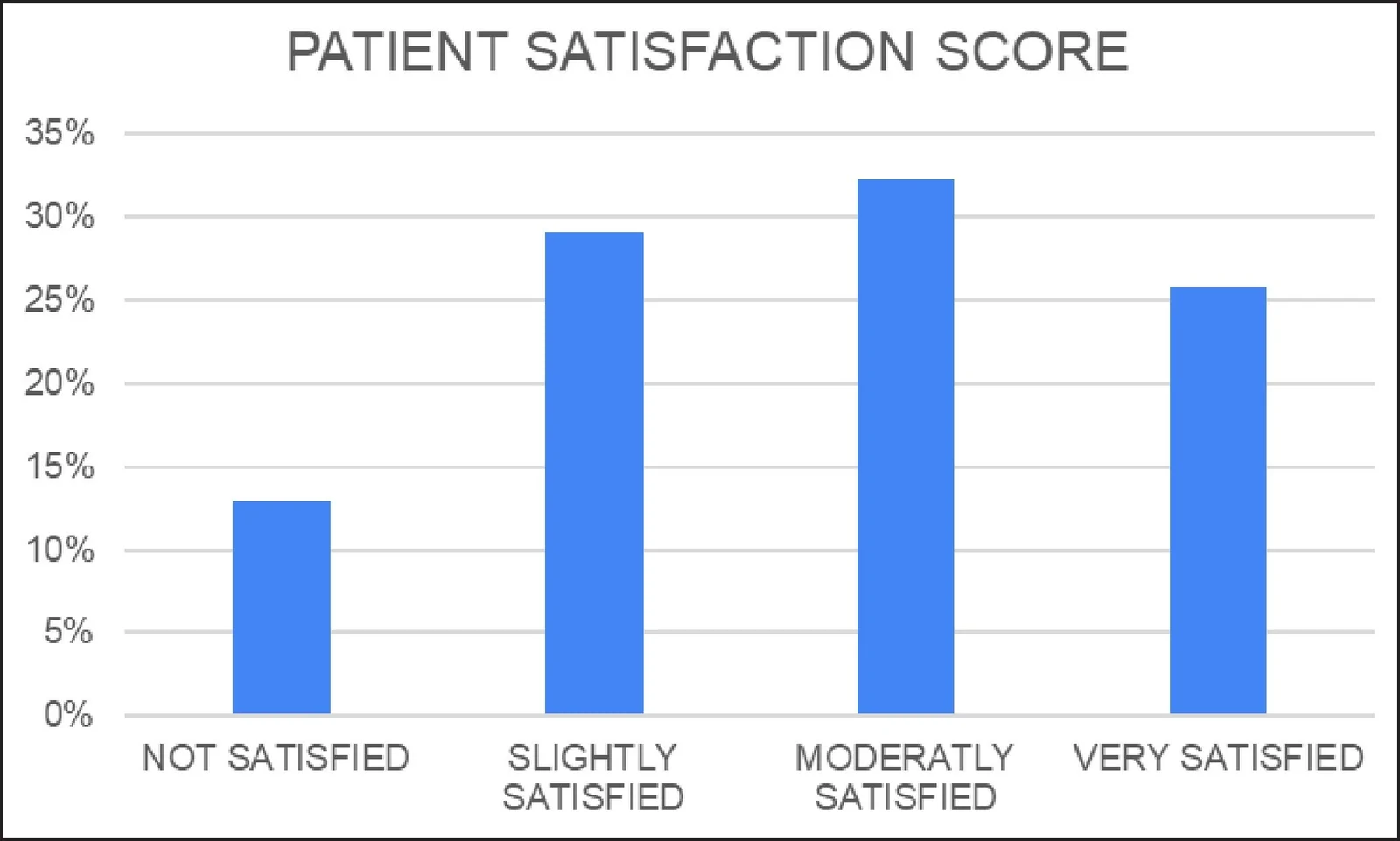
Graphical representation of patient satisfaction score.

Finally, a correlation between disease duration and treatment response was observed (Figure 11). This indicates that patients with a longer history of melasma experienced faster recovery than those with a more recent onset. This finding may suggest that earlier intervention could be associated with better outcomes, although this observation should be interpreted cautiously given the study design.

**Figure 11.**
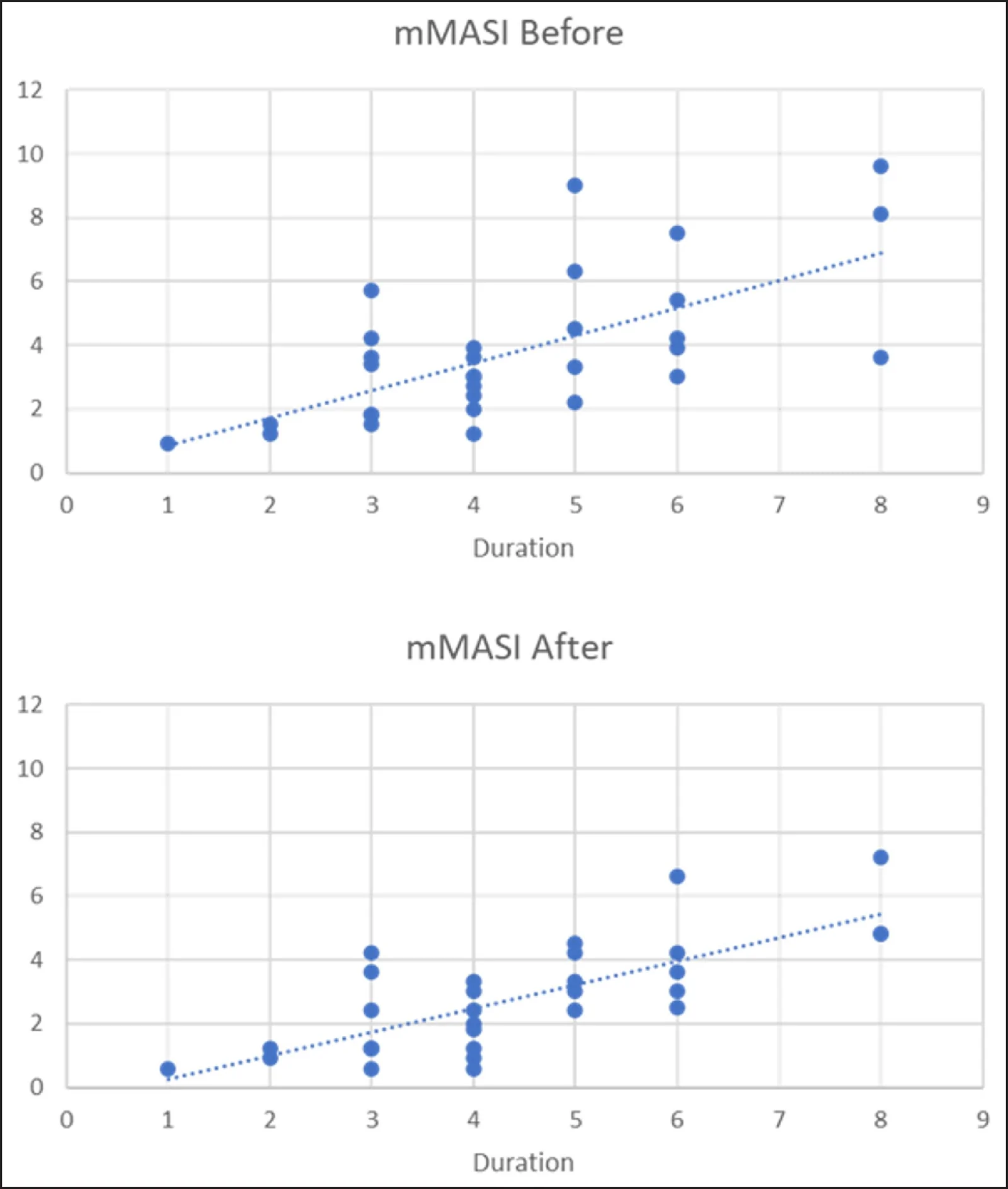
Graphical representation of the correlation between melasma duration (years) and modified Melasma Area and Severity Index (mMASI) scores measured before and after treatment.

The treatment was well tolerated. No procedures were interrupted because of pain, and no anesthesia was required. There were no major adverse effects reported during the treatment period, and all patients completed the 4 scheduled sessions without interruption. Specifically, no cases of postinflammatory hyperpigmentation, rebound hyperpigmentation, worsening of melasma, or treatment discontinuation because of intolerance were observed. Mild and transient erythema and edema were occasionally reported immediately after the procedure, resolving spontaneously within 24 to 48 hours. No persistent erythema, crusting, scaling, or other clinically significant adverse events were recorded. Treatment-related discomfort was minimal and did not require anesthesia or interruption of the procedure.

## DISCUSSION

Both qualitative and quantitative clinical data suggest a favorable 675 nm laser safety profile and a reduction in melasma severity, reporting no serious adverse effects and ensuring faster treatments as previously described compared with the previous laser system.^12^

The study results revealed that mMASI score improvement persisted for up to 3-month follow-up. Clinical photographs confirmed a decrease in hyperpigmentation and melanin deposition, as well as an overall improvement in the skin's appearance. Overall, the majority of patients reported at least a moderate level of satisfaction with the treatment outcomes.

Furthermore, a correlation between disease duration and treatment response was observed. The study analysis revealed that patients with a longer history of melasma showed a smaller degree of improvement compared with those with more recent onset. These findings suggest that early intervention may lead to better therapeutic outcomes.

Our observations are consistent with earlier published studies mentioned in the first section, and they show that the 675 nm laser appears to be helpful when treating patients with Fitzpatrick phototypes III to IV for facial melasma, with encouraging long-term outcomes, which were already reported in literature.^10^

The treatment was generally well tolerated in the present cohort, with no serious adverse events observed during the study period. Mild and transient erythema and edema were occasionally reported immediately after the procedure, resolving spontaneously within 24 to 48 hours.

The 675 nm wavelength is known to interact with melanin-containing structures and dermal collagen, as reported in previous optical, preclinical, and clinical studies.^7-14^ These characteristics provide a biological rationale for its use in pigmentary disorders such as melasma.^15^ Furthermore, histological investigations have reported selective effects in melanin-rich areas, supporting its potential role in the treatment of pigmentary disorders.^6^ Moreover, its minimal interaction with other chromophores decreases the risk of adverse effects, thereby facilitating better posttreatment management for patients. Its red spectrum emission has a weak interaction with the vascular chromophore and a high binding to collagen fibers and the melanin chromophore.^14^ By decreasing contact with other chromophores, the laser beam effectively conveys heat to the collagen fibers, causing their shrinkage and denaturation. This procedure is histologically demonstrated to accelerate the neocollagenogenesis process.^6^

Compared with other techniques used, such as chemical peels, topical, and oral medications, or a combination of these, the selective interaction of the 675 nm laser with melanin and collagen chromophores may contribute to the observed clinical improvement.

Chemical peels, topical, and systemic therapies remain standard options for melasma management, although each approach may be associated with limitations such as adverse effects, variable efficacy, and recurrence.^16^ Higher dosages of topical treatments may cause undesirable reactions, including skin irritation and peeling; they additionally have limited epidermal targeting, low stratum corneum penetration, and lengthy therapy durations.^16^

The device enables the adjustment of selected treatment parameters, including power and DOT number, whereas other parameters remain fixed depending on the modality. The density of microthermal zones can be controlled through DOT selection.^12^

By investigating the efficacy of 675 nm in these clinical scenarios, we aim to contribute to the limited body of evidence on laser therapies in skin of color and shed light on the potential benefits of this advanced technology implemented with the use of a new, faster treatment modality in controlling melasma, with minimal side effects.

Although the reduction in mMASI score observed in this study was statistically significant, the absolute change was modest (∼1 point). This finding should be interpreted in the context of the relatively low baseline severity of melasma in our cohort, suggesting that many patients presented with mild disease. In such cases, even small absolute reductions in mMASI may correspond to meaningful relative improvement. However, the overall clinical impact remains limited and should be interpreted with caution. Future studies including patients with a wider spectrum of disease severity and incorporating clinically meaningful endpoints are needed to better define the relevance of these findings.

This case series has limitations that should be considered when interpreting the findings. First, the retrospective design inherently introduces potential sources of bias, including selection bias and lack of control over confounding variables. Second, the absence of a control group or comparator treatment limits the ability to establish a causal relationship between the intervention and the observed clinical improvements. Third, the sample size, although adequate for an exploratory analysis, remains relatively limited and may not fully capture the heterogeneity of melasma presentation, particularly in terms of clinical subtypes and depth of pigmentation. In addition, the follow-up period of 3 months after the final treatment is relatively short, especially considering the chronic and relapsing nature of melasma.

However, the 3-month follow-up still provides relevant short-term clinical evidence of treatment efficacy and safety.

Furthermore, evidence on longer-term outcomes is already emerging in the literature. In particular, a recent study by Coricciati et al has demonstrated the effectiveness of the 675 nm laser in melasma treatment with follow-up extending up to 12 months, showing sustained clinical improvement over time.^10^

Future prospective, randomized controlled studies with larger sample sizes incorporating multiple follow-up time points over a longer period are needed to confirm these findings and better define the persistence of treatment effects and to define optimal maintenance strategies and the role of this treatment approach in melasma management.

Another limitation is the lack of detailed diagnostic characterization and subtype classification of melasma. Diagnosis was based on clinical evaluation without systematic use of adjunctive tools (eg, Wood's lamp or dermoscopy), and information on melasma subtypes and distribution patterns was not consistently available. These factors may influence treatment response and limit generalizability. Future studies should adopt standardized diagnostic criteria to improve patient stratification and outcome interpretation.

Finally, another limitation of this study is the lack of a validated patient-reported outcome measure; because of the retrospective design, standardized patient-reported instruments were not systematically collected. Nevertheless, a patient satisfaction score was included to provide a subjective assessment of treatment perception. Despite not being a validated scale, it provided useful complementary information regarding patients’ perception of treatment benefit in a real-world clinical setting. Future prospective investigations should incorporate validated patient-reported outcome measures to better capture the psychosocial impact of melasma and provide a more comprehensive evaluation of treatment efficacy.

## CONCLUSIONS

In this retrospective study, treatment with the 675 nm laser appeared to be effective in reducing pigmentary manifestations of facial melasma in Indian men and women, with a low incidence of side effects and simplified posttreatment management. Notably, emerging evidence in the literature suggests that these benefits may extend over longer follow-up periods, with sustained improvement reported up to 12 months. Nevertheless, larger controlled prospective studies with direct comparators are still warranted to further confirm efficacy, durability, and safety.
